# 

**Published:** 1859-01

**Authors:** 


### CONTENTS.

ORIGINAL COMMUNICATIONS— PAGE.

Report of a Case of Deformity from Fracture, successfully treated by a new method. By Daniel Brainard, M.D. .... 5
Cephalic Version. By J. H. Brower, M.D. .... 11
Hypertrophy and Dilatation of the Heart, with Hydro-Pericardium. By B. Woodward, M.D. .... 20
Case of Injury of the Brain, with an extensive Scalp Wound, and Fracture of the Skull. By W. T. Sherwood, M.D .... 23
The Preparation of the Compound Syrup of Phosphates or Chemical Food. By Dr. Phil. F. Mahla .... 27
Typhoid Fever. By B. O. Reynolds, M.D. .... 30
Poisoning by Belladonna. By B. F. Schneck, M.D. .... 32
Two Cases of Internal Injury from a Fall. By B. F. Schneck, M.D. .... 34
Report of Committee on Practical Medicine (conclusion) .... 7
Purpura Hemorrhagica; with a Report of Cases. By G.W. Phillips, M.D. 39
Valedictory Address of the President of the Illinois State Medical Society. By C. Goodbrake, M.D. .... 41

PROCEEDINGS OF MEDICAL SOCIETIES—

Annual Meeting of the Earlville Union Medical Society .... 36
Annual Meeting of the Rock Island Medical Society .... 64

BOOK AND PAMPHLET NOTICES—

The Transactions of the American Medical Association .... 51
Wilson's Human Anatomy .... 52
Diseases of the Urinary Organs .... 53

ABSTRACTS AND ITEMS. Prepared by the Junior Editor .... 56

EDITORIAL—

Preliminary Education and Medical Colleges .... 59
Acknowledgments .... 63
Prize Essays .... 63

